# Preparing for Completely Smoke-Free Mental Health Settings: Findings on Patient Smoking, Resources Spent Facilitating Smoking Breaks, and the Role of Smoking in Reported Incidents from a Large Mental Health Trust in England

**DOI:** 10.3390/ijerph13030256

**Published:** 2016-02-25

**Authors:** Harpreet Sohal, Lisa Huddlestone, Elena Ratschen

**Affiliations:** UK Centre for Tobacco and Alcohol Studies, Division of Epidemiology & Public Health, University of Nottingham, Nottingham NG5 1PB, UK; Lisa.Huddlestone@nottingham.ac.uk (L.H.); Elena.Ratschen@nottingham.ac.uk (E.R.)

**Keywords:** smoking, mental health, mental disorder, tobacco dependence, psychiatric settings, NICE PH48, smoking cessation, nicotine dependence, smoke-free policy

## Abstract

*Introduction*: Despite high smoking prevalence and excessive smoking-related morbidity and mortality among people with mental disorder compared to the general population, smoking treatment is often neglected in mental health settings. The UK National Institute of Health and Clinical Excellence (NICE) recently issued public health guidance stipulating completely smoke-free mental health settings. This project evaluated existing smoking-related practices in preparation for guidance implementation. The objectives were to: audit the recording of smoking-related information and treatment provision; explore current arrangements relating to the facilitation of patient smoking; measure staff time spent and identify costs of facilitating smoking; and explore the role of smoking in smoking-related incidents. *Methods*: A mixed-methods study was conducted across four acute adult mental health wards, accommodating 16 patients each, over six months. It included a case-note audit, on-site observations, and a qualitative content analysis of incident reports. *Results*: Smoking status was recorded for less than half of the 290 patients admitted (138, 48%). Of those, 98 (71%) were recorded as current smokers, of whom 72 (74%) had received brief smoking cessation advice. Staff spent 6028 h facilitating smoking, representing an annual cost of £131,040 across four wards. Incident reports demonstrated that smoking facilitation was often central to the cause of incidences, triggered frustration in patients, and strained staff resources***.*** *Conclusion*: The findings highlight the importance and potential of implementing completely smoke-free policies using comprehensive pathways.

## 1. Introduction

The strong links between smoking and mental disorder are well established [1]. In England, rates of tobacco smoking among people with severe mental disorder are up to three times higher than those found in the general population, with prevalence rates in hospitalised patients and patients with psychosis reaching 70% or more [2,3]. Smokers with mental disorder have been shown to have higher levels of tobacco consumption, greater levels of nicotine dependence, and to experience disproportionate levels of smoking-related morbidity and mortality [2]. Furthermore, tobacco use can directly impact psychiatric treatment, increasing the metabolism of many psychotropic medications, resulting in the need for higher medication dosages [2]. In prospective studies, smoking has been revealed to be a strong predictor of suicidal behaviour in patients, even after controlling for depressive symptoms, substance use, and previous suicidal behaviour [4].

Contrary to popular belief, people with mental disorder have been shown to be similarly motivated to quit smoking when compared to individuals without mental disorder [5,6]. Despite this and the recognised benefits of smoking cessation among people with mental disorder, which include improvements in symptoms of anxiety and depression [7], smoking cessation is widely reported to be a neglected issue within psychiatric settings [8,9,10] contributing to increased health inequalities in an already disadvantaged population [2,11]. 

Mental health Trusts across England first implemented smoke-free policies following the Health Act in 2006, stipulating that smoking was to be banned from indoor settings [12,13]. Although many Trusts attempted to extend the ban to outdoor premises, smoking on Trust premises, in the context of regularly facilitated “smoking breaks” or by granting exemptions from the policy to allow smoking in the grounds has been the norm, rather than the exception in mental health Trusts [14]. Recognising the importance of providing comprehensive and equitable support to individuals with mental disorder, the National Institute for Health and Clinical Excellence (NICE) issued public health guidance (PH48) on smoking cessation in secondary care in 2013, with one part of the guidance dedicated to mental health services [14]. In brief, the guidance called for the establishment of entirely smoke-free buildings and grounds without exceptions; the provision of comprehensive, on-site evidence-based stop smoking support; and policies which promote and support smoking cessation or temporary abstinence among patients and staff. The guidance acknowledges challenges to implementation, given the historic smoking culture in mental health settings [15,16] and was received by some with a degree of concern relating to the practicalities of implementation and enforcement. 

In many English Trusts, routine data are collected in the context of performance management (Commissioning for Quality and Innovation, CQUIN) that are specific to smoking (e.g., the routine recording of patients’ smoking status on admission, and of smoking cessation support offered to patients by staff). However, little is known about more complex implications of supporting smoking as part of daily activities on the wards, including resources spent for the facilitation of smoking breaks that occur on a regular basis [17]. This will be important to consider in the context of introducing change when implementing completely smoke-free environments—especially in terms of addressing scepticism based on arguments citing costs associated with the implementation. The current project was carried out in a large mental health Trust in England in support of preparing for the full implementation of the NICE PH48 guidance, in terms of informing the subsequent stages and decisions involved in implementing a completely smoke-free policy. Specifically, its objectives were to:

1. Audit the recording of smoking-related information (including smoking prevalence) from patient case-notes.
2. Describe current practice related to dealing with patients’ smoking (including a description of arrangements for staff-facilitated patient smoking breaks).
3. Quantify the costs associated with facilitating smoking.
4. Qualitatively explore the influence of tobacco smoking on reported incidents in acute adult in-patient mental health services.

## 2. Methods 

### 2.1. Setting and Participants

Four acute adult mental health wards (two for male, two for female patients) of one of the largest National Health Service (NHS) mental health foundation Trusts in England, accommodating up to 16 patients each, were included in the study. All wards were located at the same site, within close proximity to each other, and had outdoor courtyards. A Trust wide smoke-free policy had been implemented in 2007, however exemptions to the policy were granted on a daily basis: patients without section 17 (Section 17 leave is a section of the Mental Health Act (1983) that allows a clinician to grant a detained patient unescorted leave of absence from hospital) leave entitlement utilised the courtyard to smoke in the context of staff-facilitated smoking breaks. Patients with escorted leave were able to smoke in the presence of escorting staff, within designated areas in the grounds, or off-site in the custody of staff. Voluntary patients or patients with unescorted leave were permitted to smoke within designated areas on the grounds or to leave the site to do so. 

### 2.2. Data Collection and Analysis

A mixed methods approach was adopted, including a case-note audit of smoking status from electronic patient records to identify the extent to which patient smoking-related information was recorded (as per current CQUIN targets, *i.e*., smoking status, tobacco consumption, recording of brief smoking cessation advice); on-site observations to review smoking-related current practices/arrangements to quantify staff time to calculate cost of facilitating patient smoking breaks; and a qualitative content analysis (QCA) to explore the nature of smoking-related incidences [18]. 

#### 2.2.1. Case-Note Audit of Smoking-Related Recording

Audit standards were based on the Trust’s CQUIN targets to incentivise best practice and encourage patients to lead healthier lives in particular by supporting smokers to quit. The targets stipulated that smoking status should be recorded for 100% of patients and should include daily tobacco consumption and brief smoking cessation advice. Demographic and smoking-related data for all patients admitted from August 2014 to January 2015 were analysed descriptively at ward level. 

#### 2.2.2. Costs of Facilitating Patient Smoking 

On-site observations were conducted to quantify staff time spent facilitating smoking on each ward to derive the costs associated with this activity. On all four wards, patients without leave entitlement were permitted up to 18 smoking breaks each day, on each ward, between the hours of 6:30 a.m. and 11:30 p.m. (18 h). In order to capture the typical time invested in facilitating smoking per day, observations totalling 18 h were conducted across wards on different days and at different day times (weekends and nights excluded). The researcher observed and recorded each smoking break using a data collection log designed to record the time from which smoking-related arrangements commenced (*i.e*.**,** calling patients and distributing smoking paraphernalia) to the last patient arriving back inside the ward from the courtyard, and the door being locked by a member of staff. Any other relevant information (such as the band level of staff supervising the break) was also recorded.

To determine the cost associated with staff time spent facilitating patient smoking, the Unit Costs of Health and Social Care standard was utilised [19]. Grounded in economic theory, the document sets a standard unit cost for services and resources in health and social care [19]. The unit cost of staff per hour is more comprehensive than staff salary alone, as it is inclusive of the financial implications of all service components (*i.e*., staffing, power, maintenance, and administration). Furthermore, the ratio of direct time (work with clients) to indirect time (other activities, on a per-client basis) is factored into the calculations. 

The estimated monthly cost of staff facilitated smoking breaks were calculated by multiplying the maximum number of smoking opportunities by the median duration (in minutes) of the average break, to give the total minutes per day, per ward, and then multiplied with the relevant staff unit costs.

#### 2.2.3. Smoking-Related Incident Reports

Smoking-related incident reports from August 2014 to January 2015 were obtained from the Trusts’ reporting systems by extracting reports using relevant key terms: smoke/smoker/smoking, cigarette/s, tobacco, nicotine, lighter, nicotine replacement therapy (NRT) and e-cigarette/s. QCA was used to analyse the reports, exploring the circumstances related to the incidences, and the role smoking played in their context. The reports were read iteratively to achieve familiarisation with the raw data as a “whole” [20]. QCA was used to create meaning by identifying codes from raw data and clustering these into themes [21]. The results were validated via iterative reading and continuous reflection of the raw data and verbatim quotes were used to illustrate each of the themes with the original accounts [22]. Incident reports were read and coded independently by two researchers and where ambiguity existed between codes, these were discussed and agreement reached.

## 3. Results

Two-hundred and ninety patients were admitted to the four study wards between August 2014 and January 2015. One-hundred and forty-eight (51%) patients were male and 142 (49%) were female. Two-hundred and seventy-four (94%) patients were given a diagnosis at the time of the review (some of which were co-morbid). The most common primary diagnosis according to the International Classification of Diseases (ICD-10) was schizophrenia, which was given to 99 (36%) patients, followed by bipolar disorder (58, 21%) personality disorder (48, 17%), adjustment disorder (19, 7%), acute and transient psychotic disorder (14, 5%), recurrent depressive disorder (13, 4.7%) and post-traumatic stress disorder (7, 2%) and (16,(6%) of patients were diagnosed as “other” or “unknown” (Table 1). 

### 3.1. Case-Note Audit of Smoking-Related Recording 

Smoking status was recorded in the notes of 138 out of the 290 patients (48%). Of these, 98 (71%) reported current smoking, 11 (8%) were ex-smokers and 29 (21%) reported to have never smoked. For 152 (52%) patients, smoking status was recorded as “unknown”, which constituted an additional recording option in the system. Where recordings were missing altogether, no reason for this was given. The average daily consumption of tobacco was recorded for 92 (94%) smokers who reported to smoke a median of 20 cigarettes per day (IQR 10–20). Seventy-two (74%) smokers were recorded to have received brief smoking cessation advice and 65 (90%) were recorded to receive additional support/treatment (referral, signposting, and pharmacological treatment). Twenty-seven (20%) were provided with a leaflet, and 12 (7%) were prescribed NRT: no information regarding uptake was available (Table 2). The case-note audit indicated that the Trust was not achieving the CQUIN standard of 100% recording of smoking-related information. 

### 3.2. Costs of Facilitating Patient Smoking 

Eighteen hours of on-site observations revealed smoking break times ranged from 10 to 31 min, with a median duration of 15 min (IQR 11–15), across all wards. Patients were generally accompanied by a Healthcare Assistant (HCA), with an annual salary ranging £15,100–£19,461. Eighteen smoking breaks (with a median duration of 15 min) equated to one HCA spending an average of 270 min (4.5 h of the 7.5 h shift) per day, per ward, overseeing smoking breaks, totalling 1890 min (31.5 h) over a week. Applying the Unit Costs of Health and Social Care (£20 per hour, per HCA), the costs of facilitating smoking accumulated to £90 per day, and £630 per week. Per annum, the results indicated that staff spent 1507 h facilitating patient smoking costing in the region of £32,760 per ward. Across the four inpatient wards, this totalled £131,040 per annum, which equated to 6028 h of staff time, spent facilitating patient smoking on the Trust’s mental health wards.

### 3.3. Qualitative Content Analysis of Smoking-Related Incident Reports

Thirty-four smoking-related incident reports were extracted using the search strategy described across all wards from August 2014 to January 2015. Of those, 19 occurred on the two male wards (11 and 8, respectively), and 15 on the two female wards (11 and 4, respectively). Patient aggression (verbal and physical) towards staff was a central theme, documented in 17 (50%) of the reports. Patients absconding from wards closely followed as a common feature within nine (26%) incident reports. The QCA revealed four main themes: (1) smoking-related arrangements as a trigger of incidences; (2) tobacco use as a facilitator of undesirable behaviours; (3) smoking-related arrangements posing strains on staff resource; and (4) the utilisation of smoking as a means to mediate/de-escalate incidences. 

#### 3.3.1. Smoking-Related Arrangements as Incident Trigger (Theme 1)

Smoking-related arrangements, especially the scheduling of breaks and escorts which required patients to wait until they could smoke, frequently appeared at the centre of the emergence and escalation of reported incidences. In several instances, patients were described as displaying frustration as a result of being unable to smoke, perceiving the regulation of breaks as restrictive. Negotiating the management of urges to smoke in the context of scheduled breaks presented as a key source of conflict, sometimes triggering verbal and physical hostility towards staff:

*“Pt (patient) appeared very irritable and demanded to be let out into the courtyard for a cigarette. Pt was informed that the smoke break had finished and he must wait until the next one…Pt became very agitated, pacing the ward and punching picture frames”*.

[Male, C ward]

*“Pt became abusive and demanding of cigarette. Staff pointed the cigarette times out and Pt began throwing pots around and banging doors”*.

[Female, B ward]

On 15 (44%) occasions, the escalation triggered was sufficiently severe to require the use of *pro re nata* medication:

*“Pt threatened to smash up ward if he couldn’t have a cigarette and was verbally abusive towards staff. Verbal attempts made to distract and de-escalate Pt to no effect. Pt was given as requested medication, Lorazepam 1 mg”*.

[Male, C ward]

*“I informed Pt his room smelt as if he had been smoking…Pt shouted more verbal and racial abuse at me…Pt was moving closer to me and threatening to hit me…Pt accepted 5 mg Haloperidol and 1mg Lorazepam. This appears to have had a settling effect on him”*.

[Male, A ward]

There was evidence that absconding from the wards to forego the limitations posed by the scheduled smoking breaks was not unusual for some patients:

*“Pt absconded from the ward through the front entrance as a visitor entered. He has been unhappy at the designated smoke breaks and wished to be escorted out sooner which staff could not facilitate…Pt was given a small period of time in which to return due to the fact that he has made an earlier absconsion today for a cigarette and had returned of his volition”*.

[Male, C ward]

#### 3.3.2. Tobacco Use as a Facilitator of Undesirable Behaviours (Theme 2)

The availability of cigarettes and smoking paraphernalia during smoking breaks sometimes appeared to incite patients to engage in behaviours which could endanger themselves and/or others on the ward, through acts of concealing cigarettes and paraphernalia to smoke covertly, using paraphernalia to self-harm, conduct dangerous acts such as arson, or to treat other patients with hostility:

*“Pt was seen smoking in en-suite from a small lounge area by staff...She denied it and became abusive towards staff...She then took the lighter and cigarette from her chest area and threw it towards staff aggressively, lighter hitting staff”*.

[Female, B ward]

“*Pt was out in the courtyard area having a cigarette and she attempted to set fire to her hair with the end of her lit cigarette”*.

[Female, D ward]

*“Pt had committed arson setting fire to his curtains in his bedroom…Pt approached on the main corridor of the ward and stated that he had set his curtains on fire and that is only the start, he would be setting more fires on the war”’*.

[Male, C ward]

In some cases, stealing other patients’ cigarettes led to hostile and violent behaviour:

*“Pt wanted a cigarette...She got frustrated and started shouting and swearing...She stormed into (another) patient’s room…demanding a cigarette waking her up in a hostile manner, staff intervened…Pt stormed into the office and grabbed one of the patient’s cigarettes”*.

[Female, D ward]

*“Pt was having a cigarette break when he took another patient’s cigarettes, staff tried to take the cigarettes asking him to hand them over, Pt refused…and lit one of the cigarettes. Staff moved in and tried to remove them at this point, Pt lashed out with his arm and staff moved into passive…Pt was kicking his legs between staff legs…he managed to throw me (staff member) to the ground, where I hit my head on contact with the ground”*.

[Male, C ward]

#### 3.3.3. Smoking-Related Arrangements Posing Strains on Staff Resource (Theme 3)

Although patients without granted leave from wards were permitted to smoke within the allocated courtyard during scheduled break times, supervised by one member of staff, patients deemed “at risk” required one-to-one support, which during regular smoking breaks strained staffing levels, contributing to a staff shortage. Consequently, a report was filed concerning an unsafe working environment due to low staffing levels on the ward:

*“Pt came out for a cigarette break in the courtyard...Pt went to the far end of the courtyard and tied a shoe lace around her neck and then onto the railings...Ligature knife was used firstly to cut shoe lace from railing…Decision made Pt is to be taken 1:1 only for smoke breaks”*.

[Female D ward]

*“Pt is currently on high observations eyesight level after several attempts of suicide...Pt was trying to scale the fence and was being quite aggressive with staff who were trying to stop her. It has been snowing and it was very slippery trying to hold the Pt…Plan was made for Pt to continue to have her cigarette breaks…Pt would need to be supported by two staff members”*.

[Female, B ward]

#### 3.3.4. Utilisation of Smoking as Means to Mediate/De-escalate Incidences (Theme 4)

Reports demonstrated that staff sometimes facilitated smoking outside of scheduled breaks to “calm” patients in heightened acute states. However, this method of conflict resolution appeared to unintentionally reinforce behaviours which triggered the initial incident, and appeared to mediate unfavourable behaviours in patients:

*“Pt was escorted to the courtyard for a cigarette break to attempt to de-escalate his frustrations”*.

[Male, A ward]

*“Staff supported her to have a cigarette but informed her that it was unacceptable to go in patient’s room whilst patients are asleep demanding cigarette, she was also told that it is not acceptable to storm into the office and grab other patient’s cigarettes. Patient was vile, verbally aggressive, rude, loud and feisty”*.

[Female, D ward]

Whilst staff intended to offer smoking breaks outside of the scheduled times in an effort to de-escalate situations, it was reported to have led to other problems such as patients seizing the opportunity to abscond from Trust premises:

*“Due to Pt’s need to have cigarette she was let out about 20:45 for a cigarette break. Pt absconded over the fence. At 21:00 Pt was returned to the ward by the police in passive restraint”*.

[Female, B ward]

Noticeably, despite smoking mediating certain negative behaviours, successful resolution post-conflict were reported by staff to end with patients engaging in smoking:

*“He went for a cigarette and then retired to his bed space”*.

[Male, A ward]

*“Pt eventually calmed himself and accepted his medication then utilised the courtyard for a cigarette”*.

[Male, C ward]

## 4. Discussion

This mixed-method study demonstrates high patient smoking prevalence; low compliance with targets set for the provision of tobacco dependence treatment for smokers; substantial yearly (£131,040) investment to facilitate patient smoking; and a range of negative implications associated with permitting patient smoking in the context of complex social interactions. 

### 4.1. Costs of Facilitating Smoking

We found that a disproportionate amount of resource was spent facilitating smoking breaks, utilising 60% (4.5 h, per day) of an HCA full-time equivalent, over £30,000 per ward per year, and potentially impacting on staff opportunities to engage patients in therapeutic activities. It should be noted that the resources measured did not factor in other related arrangements such as distributing/collecting smoking paraphernalia and dealing with related incidences, which, if included, would increase the expense of resources even further. A further consideration is that the calculations reported here are based on one HCA facilitating each smoking break. However, these figures may be an underestimation of the true costs: as indicated in the incident reports, if patients were deemed at risk, up to two HCA were required to supervise smoking breaks thus further increasing the financial burden to Trusts. Additionally, the use of HCA is widespread within UK’s psychiatric settings, however within other countries nursing staff may be the primary staffing group to supervise smoking breaks therefore associated costs may be quite variable—and possibly higher. 

Research has demonstrated that the implementation of a smoke-free facility can save valuable time for staff, as providing patients with smoking cessation advice, pharmacological treatment and behavioural counselling requires less time to that of supervising smoking [23,24]. Economic analyses have shown smoking cessation interventions to be one of the more cost-effective healthcare interventions available, with combination therapy being more cost-effective than brief advice or counselling alone [25,26]. The time and costs saved when eliminating patient smoking and smoking breaks could be re-invested to support smoking cessation and greater delivery of therapeutic activities [27]. Within inpatient settings, it has been shown that greater staff-patient engagement and patient participation in therapeutic activities is linked to improved clinical outcomes for individuals with mental disorder, whilst also being cost-effective [28].

### 4.2. Smoking-Related Recording and Support Pathways

Failure to record accurate and relevant smoking-related information limits opportunities to address smoking appropriately in mental health settings and potentially compromises equity of care amongst smoking and non-smoking patients in smoke-free environments. Where patients were recorded as smokers in our study, there was relatively high compliance with the delivery of brief advice and the offer of support to quit, highlighting the importance of identifying smokers. Unhelpfully, the option to record smoking status as “unknown” was available, allowing for ambiguity. In the context of implementing a full smoke-free policy, data collection via electronic patient recording systems should be regularly reviewed and if necessary, adapted.

Despite being one of the most effective forms of tobacco dependence management, NRT was found to be infrequently prescribed with a low uptake. Most commonly, patients were given a leaflet regarding smoking cessation. In a population with a higher than average tobacco dependence [2], symptoms of withdrawal are likely to be experienced intensely. The near absence of NRT to manage patient withdrawal between smoking breaks and overnight highlights inadequacies in managing this clinical issue. These findings reflect that of international research which showed that mental health nurses and medical staff did not provide adequate nicotine dependence treatment to smokers in a psychiatric setting [29]. Other international research also conducted in a psychiatric facility, demonstrated that the provision of nicotine dependence treatment was rarely recorded, with patients receiving pharmacological treatment at discharge and not at admission [30]. Training and resources for staff to maximise the uptake of smoking cessation offers, that include behavioural, as well as pharmacological support, are likely to play a central role in the effort to implement NICE guidance PH48. 

### 4.3. Smoking-Related Complexities: Incidents

The QCA revealed that smoking-related arrangements appeared integral to the cause of incidences and negatively affect staff-patient interactions, as evidenced when staff are required to enforce the timings of smoking breaks. Patients deem the arrangements and frequency of breaks as inadequate, which may constitute a pathway for challenging behaviour, including aggression and hostility, and attempts of absconding and arson. Furthermore, there appears to be a strain placed on staff when one-to-one supervision for at risk patients is required during smoking breaks, thus contributing to a staff shortage. 

The issue is perpetuated by the inconsistent enforcement of the smoking policy by staff in order to de-escalate incidents. There is a concern that, where hostile behaviour is de-escalated by providing a smoking break, it is reinforced—especially when the hostility was originally triggered by the inability to smoke at leisure. In our study, *pro re nata* medication was commonly used as a means of de-escalation, particularly when patients’ smoking-related requests could not be met and verbal de-escalation was unsuccessful. Although it is not possible to establish linear causal links between the regular facilitation of smoking and the need for use of *pro re nata* medication, we feel it is likely to assume that inconsistent management of smoking and smoke-free regulations, including the facilitation of regular breaks and the availability of cigarettes and paraphernalia on ward premises triggers the occurrence of escalations and incidents. This is especially likely when considering the complexities involved in social interactions related to smoking, and the use of cigarettes as a tool for negotiation and de-escalation, and as a currency [8].

In view of the complexities involved in the maintenance of smoking on our study wards, and the central role smoking-related arrangements and smoking paraphernalia played in incidences, we argue that the creation of completely smoke-free environments would be beneficial as evidenced elsewhere [31]. Smoke-free environments would also support a decrease in triggers for violent incidents, this would be in line with a review of 26 international studies exploring smoking bans in inpatient settings, highlighting that despite staff anticipation to the contrary, there were fewer problems reported post-implementation, with no reported increase in patient aggression or in administration of *pro re nata* medication [32]. Similarly, in a UK high-security setting, there was no evidence of a statistically significant increase in incidences post-implementation [33]. Lastly, an Australian survey found that staff reported patient care to be less challenging after a completely smoke-free environment was implemented [34].

### 4.4. Limitations

Our findings are limited by some methodological considerations, especially the collection of data from a small sample of four acute adult mental health wards using observational methods only, potentially introducing common biases. Whilst the current research did not find evidence of therapeutic interaction during observed smoking breaks, it has been reported elsewhere [8]. However, other findings have demonstrated that the therapeutic relationships between patients and staff were not negatively affected [35]. We are aware that smoking-related arrangements vary across Trusts in England, with some having already implemented the complete smoke-free guidance in full. A further limitation consists in the circumstance that our findings relate to one type of unit only (acute adult mental health), whereas there is evidence to highlight the importance of recognising idiosyncrasies across different unit types [36]. However, most Trusts are presently preparing for implementation, and some scepticism with regard to the practicalities of the endeavour remain; as does the facilitation of smoking as a norm at this transitional stage. Given the size of the study Trust and its patient population that is broadly representative of the population of people with severe mental illness, we believe that our results are relevant and applicable to Trusts across the country, and of interest also to the international mental health community.

## 5. Conclusions 

Our findings highlight the probable benefits of making mental health inpatient settings completely smoke-free. They also demonstrate that successful implementation of NICE guidance PH48 is likely to depend on the consistent collection of suitable smoking-related information, and the provision of training and resources to enable staff to support smokers adequately and to promote change. Importantly, they highlight the opportunity to re-invest resources currently expended on facilitating smoking in the interest of increasing therapeutic activities for mental health inpatients. Research evaluating the effects of establishing completely smoke-free mental health Trusts, measuring relevant clinical and organisational outcomes, will be an important component of continued progress in this area.

## Figures and Tables

**Table 1:** Summary of participant characteristics.

Patient Characteristic (*n* = 290)		Frequency (%)
Accommodation	*Ward A*	63 (22)
	*Ward B*	77 (27)
	*Ward C*	85 (29)
	*Ward D*	65 (22)
Gender	*Male*	148 (51)
	*Female*	142 (49)
Primary diagnosis	*Schizophrenia*	99 (36)
	*Bipolar disorder*	58 (21)
	*Personality disorder*	48 (18)
	*Adjustment disorder*	19 (7)
	*Acute and transient psychotic disorder*	14 (5)
	*Recurrent depressive disorder*	13 (5)
	*Post-traumatic stress disorder*	7 (2)
	*Other/Unknown*	16 (6)

**Table 2:** Summary of recording in relation to the identification and treatment of tobacco dependence.

Recording in Relation to the Identification of Smokers			
Audit Standard to be Achieved in 100% of Cases			Frequency (%)
Patient questioned in relation to smoking status (*n* = 290)		*Yes*	138 (48)
		*No*	152 (52)
**Recording in Relation to the Treatment of Tobacco Dependence**			
**Audit Standard to be Achieve in 100% of Cases**			**Frequency (%)**
Level of tobacco consumption recorded (*n* = 98)		*Yes*	92 (94)
		*No*	6 (6)
Delivery of brief smoking cessation advice (*n* = 98)		*Yes*	72 (73)
		*No*	26 (27)
Support or treatment offered (*n* = 72)		*Yes*	65 (90)
		*No*	9 (10)
**Treatment or Support Offered to Patients for Tobacco Dependence**			**Frequency (%)**
Type of treatment or support (*n* = 65)	*Patient provided with a leaflet*		27 (42)
	*Patient signposted to support*		6 (9)
	*Referral to stop smoking service*		20 (31)
	*Pharmacotherapy prescribed*		12 (18)

## Abbreviations

The following abbreviations are used in this manuscript:

NICE: National Institute of Clinical Excellence
CQUIN: Commissioning for Quality and Innovation
NHS: National Health Service
QCA: Qualitative Content Analysis
NRT: Nicotine Replacement Therapy
HCA: Healthcare Assistant
ICD-10: International Classification of Diseases
